# Alactic base excess is not a sensitive or specific diagnostic tool for outcome in horses with colic

**DOI:** 10.3389/fvets.2025.1618304

**Published:** 2025-08-15

**Authors:** Corinne E. Crosby, Annette O’Connor, Amelia S. Munsterman

**Affiliations:** Department of Large Animal Clinical Sciences, Michigan State University College of Veterinary Medicine, Michigan State University, East Lansing, MI, United States

**Keywords:** horse, exploratory celiotomy, alactic base excess, colic, acute abdomen

## Abstract

**Introduction:**

Colic is a significant source of morbidity and mortality in horses, and no single biomarker can distinguish surgical from medical colic or predict mortality. Alactic base excess (ABE) has shown an association with mortality and renal insufficiency in humans but has not been evaluated in veterinary species. The purpose of this study was to determine the value of ABE as a diagnostic tool for horses treated for gastrointestinal disease.

**Methods:**

This retrospective case series evaluated horses admitted for acute gastrointestinal disease over a 5-year period (2019–2024). Signalment, physical examination findings, venous blood gas analysis results, packed cell volume and total solids on admission, findings in cases undergoing exploratory celiotomy, and outcome were collected from the medical record. Variables were evaluated individually, and by multivariate analysis for sensitivity and specificity to differentiate medical from surgical colic, strangulating versus non-strangulating lesions, and between survivors and non-survivors after surgical intervention. Optimal Youden cut-off values and area under the receiver operating curve (AUC) were calculated.

**Results:**

Five hundred and thirty eight horses met the inclusion criteria. Multiple presentations by the same horse were each counted as individual presentations, resulting in 585 admissions. While ABE was higher in surgical cases that did not survive (*p* = 0.029), it did not discriminate between medical and surgical colic or between survivors and non-survivors as a sole diagnostic or multivariate modality. The best predictor of medical or surgical treatment was serum glucose levels (AUC = 0.76, Se = 0.76, Sp = 0.67). Serum glucose levels were also the best predictor of strangulating versus non-strangulating lesions (AUC = 0.81, Se = 0.67, Sp = 0.83). The model for survival after surgery included age and serum L-lactate (AUC = 0.76, Se = 0.73, Sp = 0.69).

**Discussion:**

Elevations in alactic base excess values in surgical cases that did not survive may indicate compensation for hyperlactatemia with complex metabolic derangements. Our investigation supports the use of hyperglycemia and hyperlactatemia as markers of severity for horses with gastrointestinal disease.

## 1 Introduction

Colic is a significant source of morbidity in horses, with 10% of horses diagnosed with colic in their lifetime, and 16% of those experiencing repeat episodes (1). In addition, it is reported that half of horses with abdominal pain presented to referral institutions for additional evaluation and therapy required surgical intervention (2). Horses experiencing colic present with varying degrees of dehydration and often have received nephrotoxic drugs in the form of non-steroidal anti-inflammatories in an attempt to manage their discomfort. Consequently, acute renal injury may result and influence not only therapeutic recommendations, but prognosis as well (3). A swift and informed decision regarding treatment is beneficial to the horse and owner. To date, no single biomarker has been shown to provide a definitive diagnosis or outcome (4).

In the last 6 years, the concept of alactic base excess has been introduced in human medical literature in an effort to better understand metabolic derangements, specifically lactic acidosis and its role in critically ill patients. Alactic base excess (ABE) differentiates between metabolic acidosis secondary to hyperlactatemia from that caused by an increase in fixed acids, otherwise known as unmeasured strong anions (5). These fixed acids increase in the presence of renal tubular dysfunction and may identify the presence of subclinical organ failure. In humans, a significant correlation has been shown between septic hyperlactatemic patients with a negative serum ABE (< −3 mmol/L) and overall mortality (5, 6). Patients who exhibit a neutral ABE (−3 to +4 mmol/L) indicate sufficient renal compensation for increased lactic acid and maintain a neutral pH (7). For patients with an elevated alactic base excess (> 4 mmol/L), an alkalosis may be present suggesting the kidneys have adequately compensated for metabolic acidosis (7). To the author’s knowledge, the concept of ABE has not been investigated in veterinary literature leaving its role as a prognostic indicator for horses with colic unknown.

The purpose of this retrospective study was to evaluate the role of ABE in medical and surgical management of horses with colic and evaluate its utility in predicting outcomes including the need for surgical intervention, the presence of a strangulating lesion, and mortality after surgery. The primary objective was to determine if individual variables from the signalment, physical exam and bloodwork, including a serum blood gas, were associated with prediction of outcomes including: medical versus surgical treatment of colic, the presence of a strangulating (SL) versus non-strangulating (NSL) intestinal lesion, and survival to discharge in horses that underwent an exploratory laparotomy. The secondary purpose of the study was to explore the variables that could be used as either a sole determinant or combined as a diagnostic tool for the same three outcomes. The hypothesis was that ABE would be lower in horses with surgical compared to medical lesions, in strangulating versus non-strangulating obstructions, and in horses that did not survive to discharge after surgical intervention.

## 2 Materials and methods

The study was designed as a single-gate retrospective study, with all eligible horses enrolled. The sampling method was consecutive, as horses were enrolled as they presented to the hospital. The reference test was the observed outcome, e.g., did the horse receive medical or surgical treatment. For strangulating lesions (SL), horses were categorized as SL if diagnosed with one of the following: small intestinal strangulations including pedunculated lipomas, hernias, volvulus, mesenteric rents, diaphragmatic hernias; large intestinal volvulus or incarceration; cecal volvulus; descending colon volvulus or pedunculated lipoma. Survival after surgical intervention was defined as horses that were discharged alive.

### 2.1 Participants

Medical records of horses referred to the Michigan State University Large Animal Hospital from 2019 to 2024 were reviewed to identify horses greater than 1 year of age admitted for colic. All breeds were eligible. A venous blood gas obtained with a capped, manually heparinized standard syringe on admission was required for inclusion in the study. Horses with colitis, peritonitis and reproductive disease were excluded. Horses with gastrointestinal rupture at the time of admission, during hospitalization or at the time of initial abdominal exploration were also excluded.

### 2.2 Clinical data and tests recorded from the medical records

Information identified from the medical record included date of admission and discharge, age, breed, sex and initial clinical examination parameters including heart rate, respiratory rate and rectal temperature. A packed cell volume (PCV) (%) and serum total solids (TS) (g/dl) were obtained concurrently with the venous blood gas and recorded along with venous blood gas parameters including pH, pCO_2_, base excess-extracellular fluid (BE-ECF), L-lactate, bicarbonate (HCO_3_), sodium, potassium, chloride, ionized calcium and magnesium, anion gap (AG), creatinine, blood urea nitrogen (BUN), and glucose. Values for alactic BE (ABE), strong ion difference (SIDa), and A total (Atot) were calculated from admission values. ABE was calculated as:


ABE=BE−ECF+L−lactate mmol/L

SIDa was calculated as:


SIDa=(Na+K)−(Cl+L−lactate mmol/L)

Atot was calculated as:


$$\text{Atot}=0.2^{*}\;\mathrm{TS}\;(g/L)$$

Blood gas concentrations were measured using venous blood collected with a heparinized syringe from a jugular vein on a bench-top chemistry analyzer (Nova Prime Vet by Nova Biomedical) Packed cell volume and total solids were obtained from the same sample or using a separate heparinized syringe by centrifugation of a hematocrit tube and use of a hand-held refractometer. Reference ranges are provided in Supplementary material 1 (33).

### 2.3 Data categorization

The diagnosis was recorded, and horses grouped by treatment into medical only, or those with surgical intervention. Non-survivors that did not have the diagnosis confirmed with surgery or necropsy were excluded. Horses treated medically that survived to discharge were assumed to have a non-strangulating obstruction. Surgical cases were identified as strangulating or non-strangulating, and further categorized by intestinal segment (small intestinal, large intestinal, cecum and small colon) and specific lesion. The outcome of surgical cases was divided into those that were euthanized or died in hospital, and those that survived to discharge.

### 2.4 Data analysis

Categorical study population characteristics were described with counts and percentages. Descriptive analysis for continuous variables was provided by computation of the medians and interquartile ranges (IQR) and number missing for those with a Shapiro Wilk test; other variables are represented as mean (SD). Statistical analyses were performed using commercial statistical software (R Core Team v2024.12.1), with statistical significance defined as *p* < 0.05.

For the primary objective, univariate analysis was performed with a non-paired Wilcoxon rank test for differences in the continuous variables (age, rectal temperature, heart rate, respiratory rate, PCV, TS, pH, pCO_2_, BE-ECF, L-lactate, ABE, HCO_3_, sodium, potassium, ionized calcium, ionized magnesium, chloride, anion gap, strong ion difference, creatinine, BUN, Atot, and glucose) across the outcomes. The outcomes were identified as medical or surgical treatment, strangulating or non-strangulating intestinal lesions, and survival to discharge after surgery. For categorical variables (breed, sex) differences in the frequency across the outcome groups were evaluated using a Chi-squared test. A receiver operator characteristic curve (ROC) analysis was performed (R package “pROC”) for individual variables to determine the area under the curve (AUC) for prediction of (1) medical versus surgical, (2) strangulating versus non-strangulating and (3) survival and mortality in surgical cases. An area under the curve above 0.8 was considered clinically useful (8).

The secondary objective aimed to assess the diagnostic value of the metrics measured at presentation to differentiate between cases that (1) required medical versus surgical treatment, (2) were strangulating versus non-strangulating lesions, and (3) the survival or mortality in surgical cases. The potential prediction variables available to all models were age, sex, pH, BE-ECF, L-lactate, ABE, HCO_3_, sodium, potassium, ionized calcium, ionized magnesium, chloride, anion gap, strong ion difference, creatinine, blood urea nitrogen, glucose and PCV. The following variables were excluded due to missing data: temperature, heart rate, respiratory rate, total solids, Atot, and pCO_2_.

A lasso method of variable selection was used (9). The package used was a cross-validation approach using the R package” gimnet “(set seed at 123). Alpha was set to 1, and all x variables were scaled. For each model, the predicted outcome was obtained using the minimum lambda to predict the y. The ROC curve was created using the observed and predicted y (R package” pROC“) (10). The cut-off for the predicted y was chosen based on maximizing the Youden index. The selected variables, the confusion matrix, precision, accuracy, sensitivity, specificity, and corresponding 95% confidence intervals are presented, with the ROC curves.

## 3 Results

### 3.1 Study population

During the 5-year study period, a total of 538 horses admitted with a presenting complaint of colic and had a venous blood gas performed at the time of admission. Of these, 47 horses (8.7%) were admitted on more than one occasion (range 1–5), with 36 (6.7%) horses admitted twice, 2 horses (0.37%) admitted 3 times, and one horse each (0.19%) admitted 4 and 5 times, respectively. Each admission was treated separately for statistical analysis for a total of 585 admissions. There were 211 (39.2%) mares, 298 (55.3%) geldings and 30 stallions (5.6%). Ages ranged from 1 to 33 years (median = 12 years). Breeds included Quarter/stock horses and Paints (*n* = 153; 24.4%), Warmbloods (*n* = 111; 20.6%), Thoroughbreds (*n* = 57; 10.6%), Arabians (*n* = 50; 9.3%), American gaited horses (e.g., American Saddlebred, Morgan, Tennessee walking horse, Missouri fox trotter, Rocky Mountain, Dutch harness horse, *n* = 38; 7.1%), ponies (*n* = 24; 4.5%), draft breeds (*n* = 21; 3.9%), American miniatures (*n* = 20; 3.7%), Friesians (*n* = 16; 3.0%), Standardbreds (*n* = 14; 2.6%), Spanish gaited horses (e.g., Andalusian, Lusitano, Paso Fino, *n* = 13; 2.4%) and mixed breeds (22; 4.1%).

The data was not normally distributed based on the results of the Shapiro Wilk test, and non-parametric statistical analysis was performed. Overall, the median heart rate was 48 bpm (range: 24–132 bpm), respiratory rate was 20 brpm (range: 8–80 brpm) and temperature was 99.8°F (range: 94.2–103.6°F). There was not sufficient evidence to reject the null hypothesis that distribution of mean age and frequency of breed were the same for all the outcomes (*p* > 0.07).

The original dataset of 585 admissions were divided into 369 (63.1%) cases treated medically and 216 (36.9%) that were diagnosed with a surgical lesion and underwent an exploratory laparotomy. A total of 130 (61.1%) of the 216 surgical cases were strangulating lesions. Of the surgical cases, 125 (57.9%) survived to discharge with 64 (51.2%) of these horses diagnosed with strangulating lesions. There were 91 surgical cases that did not survive, and 22 (24.1%) were strangulating.

### 3.2 Medical and surgical treatment

When comparing medical and surgical cases by individual clinical variables using a univariate analysis, vital parameters were statistically different between groups (*p* < 0.001). The median heart rate and respiratory rate for both were increased outside of reference values, and higher in the surgical cases (Table 1). The L-lactate was higher and above the reference range in the surgical patients (median = 1.8 (IQR = 2.4) mmol/L) versus medical cases (median = 1.1 (IQR = 1.0) mmol/L; *p* < 0.001), along with a higher anion gap (surgical median = 13.0 (IQR = 9.5) mmol/L versus medical median = 10.5 (IQR = 7.0) mmol/L; *p* = 0.002). The pH was lower for surgical patients compared to medical cases (median = 7.42 (IQR = 0.07) versus median = 7.44 (IQR = 0.04); *p* < 0.001) combined with a lower chloride (median = 102 (IQR = 5.0) versus median = 103 (IQR = 3.0); *p* = 0.001) and strong ion difference (median = 42.6 (IQR = 4.65) versus median = 43.4 (IQR = 4.1); *p* = 0.024). However, the pH was within reference range for both groups. Both medical (median = 5.7 (IQR = 0.4) mg/dL) and surgical cases (median = 5.5 (IQR = 0.6) mg/dL) demonstrated hypocalcemia, although the remainder of the electrolytes were within reference ranges. The renal values were clinically identical despite a statistical difference observed in creatinine (surgical median = 1.2 (IQR = 0.4) mg/dL versus medical median = 1.2 (IQR = 0.4) mg/dL; *p* = 0.003), and creatinine and BUN were within reference ranges for both groups. Surgical patients demonstrated hyperglycemia (median = 139.5 (IQR = 83) mg/dl), which was significantly higher than medically treated horses (median = 100 (IQR = 32) mg/dl; *p* < 0.001).

**Table 1:** Univariate analysis for clinical variables associated with medical or surgical treatment.

Variable	Non-surgical Median (IQR) (*N* = 369)	Surgical Median (IQR) (*N* = 216)	Wilcoxon Test *p*-value
Age (years, *N* = 585)	12 (10)	12.5 (10.5)	0.133
Temperature (°F, *N* = 534)	99.9 (1.2)	99.5 (1.9)	<0.001
Heart rate (beats/min, *N* = 558)	48(12)	54 (28)	<0.001
Respiratory rate (respirations/min, *N* = 546)	20 (8)	24 (12)	<0.001-
PCV (%, *N* = 510)	38 (8)	38 (9)	0.471
Total solids (g/dl, *N* = 509)	6.7 (0.7)	6.8 (1.15)	0.255
pH (*N* = 585)	7.44 (0.04)	7.42 (0.07)	<0.001
pCO_2_ (mmHg, *N* = 584)	39.4 (8.7)	40.1 (10.3)	0.347
Base excess-ECF (mmol/L, *N* = 583)	2.70 (6.80)	1.30 (9.45)	0.062
Lactate (mmol/L, *N* = 585)	1.1 (1.0)	1.8 (2.4)	<0.001
Alactic base excess (mmol/L, *N* = 585)	4.1 (6.8)	4.45(8.5)	0.624
HCO_3_ (mmol/L, *N* = 585)	26.9 (6.6)	26.1 (8.95)	0.157
Sodium (mmol/L, *N* = 585)	137 (3.0)	137 (3.0)	0.242
Potassium (mmol/L, *N* = 585)	3.6 (0.5)	3.5 (0.4)	0.716
Ionized calcium (mg/dL, *N* = 585)	5.7 (0.4)	5.5(0.6)	<0.001
Ionized magnesium (mg/dL, *N* = 585)	1.0 (0.3)	1.0 (0.3)	0.76
Chloride (mmol/L, *N* = 584)	103 (3)	102 (5)	<0.001
Anion Gap (mmol/L, *N* = 576)	10.5 (7.0)	13.0 (9.5)	0.002
Strong ion difference (mmol/L, *N* = 584)	43.4 (4.1)	42.6 (4.65)	0.024
Creatinine (mg/dl, *N* = 580)	1.2 (0.4)	1.2 (0.4)	0.003
Blood urea nitrogen (mg/dl, *N* = 585)	15 (5.0)	16 (6.0)	0.011
Atot (*N* = 5.9)	1.34 (0.14)	1.36 (0.22)	0.206
Glucose (mg/dl, *N* = 582)	100 (32)	139.5 (83)	<0.001

### 3.3 Strangulating versus non-strangulating lesions

Analysis of horses with strangulating lesions noted the median age was higher (median = 14.5 (IQR = 9) years) than those with non-strangulating lesions (median = 11 (IQR = 10) years; *p* < 0.001) (Table 2). Tachycardia and tachypnea were noted in both groups, which was higher in horses with a strangulating lesion (median = 56 (IQR = 32) bpm versus median = 48 (IQR = 16) bpm; *p* < 0.001). Hyperlactatemia was observed in horses with strangulating lesions (median = 1.9 (IQR = 2.35) mmol/L versus median = 1.1 (IQR = 1.25) mmol/L; p < 0.001) with a lower pH (median = 7.42 (IQR = 0.07) mmol/L versus median = 7.43 (IQR = 0.04) mmol/L; *p* < 0.001); however, pH was within reference range for both groups. Hypocalcemia was present in both groups and was lower in strangulation cases (surgical median = 5.4 (IQR = 0.5) mg/dL versus medical median = 5.7 (IQR = 0.4) mg/dL; *p* < 0.001) along with a lower chloride (median = 101.5 (IQR = 5.0) mmol/L versus median = 103 (IQR = 3.0) mmol/L; p < 0.001). Electrolytes other than ionized calcium were within reference range. The BUN was statistically different between groups (surgical median = 16 (IQR = 5.5) mg/dL versus medical median = 15 (IQR = 5.0) mg/dL; *p* = 0.025), but clinically similar and not out of reference range. A hyperglycemia (median = 153 (IQR = 88) mg/dl) was noted in horses with intestinal strangulation, but was within normal limits in horses without strangulation (median = 103 (IQR = 36) mg/dl; *p* < 0.001).

**Table 2:** Univariate analysis for clinical variables associated with strangulating lesions.

Variable	Non-strangulating median (IQR) *N* = 453	Strangulating median (IQR) *N* = 132	Wilcoxon test *P-*value
Age (years, *N* = 585)	11 (10)	14.5 (9)	<0.001
Temperature (°F, *N* = 534)	99.9 (1.3)	99 (1.9)	<0.001
Heart rate (beats/min, *N* = 558)	48(16)	56 (32)	<0.001
Respiratory rate (respirations/min, *N* = 546)	20 (9)	24 (14)	0.001
PCV (%, *N* = 510)	38 (8)	38 (10)	0.165
Total solids (g/dl, *N* = 509)	6.7 (0.9)	6.7 (0.7)	0.369
pH (*N* = 585)	7.43 (0.04)	7.42 (0.07)	<0.001
pCO_2_ (mmHg, *N* = 584)	39.3 (8.95)	41.0 (9.85)	0.042
Base excess-ECF (mmol/L, *N* = 583)	2.3 (7.3)	1.95 (9.55)	0.684
Lactate (mmol/L, *N* = 585)	1.1 (1.25)	1.9 (2.35)	<0.001
Alactic base excess (mmol/L, *N* = 585)	4.0 (7.3)	4.9 (7.95)	0.085
HCO_3_ (mmol/L, *N* = 585)	26.8 (6.9)	26.5 (9.0)	0.965
Sodium (mmol/L, *N* = 585)	137 (3.0)	137 (3.0)	0.508
Potassium (mmol/L, *N* = 585)	3.6 (0.5)	3.5 (0.4)	0.408
Ionized calcium (mg/dL, *N* = 585)	5.7 (0.4)	5.4 (0.5)	<0.001
Ionized magnesium (mg/dL, *N* = 585)	1.0 (0.3)	1.0 (0.3)	0.654
Chloride (mmol/L, *N* = 584)	103 (3.0)	101.5 (5.0)	<0.001
Anion gap (mmol/L, *N* = 576)	11.0 (7.0)	13.0 (10.0)	0.075
Strong ion difference (mmol/L, *N* = 584)	43.2 (4.3)	42.9 (4.4)	0.360
Creatinine (mg/dl, *N* = 580)	1.2 (0.4)	1.2 (0.4)	0.169
Blood urea nitrogen (mg/dl, *N* = 585)	15 (5.0)	16 (5.5)	0.025
Atot (*N* = 5.9)	1.34 (0.18)	1.34 (0.14)	0.380
Glucose (mg/dl, *N* = 582)	103 (36)	153 (88)	<0.001

### 3.4 Surgical outcomes

Surgical non-survivors were older (median = 15 (IQR = 9) years) than survivors (median = 12 (IQR = 10) years; *p* = 0.01), with a higher heart rate (median = 60 (IQR = 32) bpm vs. median = 52 (IQR = 22) bpm; *p* = 0.002) and respiratory rate (median = 24 (IQR = 12) vs. median = 20 (IQR = 12); *p* = 0.038) (Table 3). Both survivors and non-survivors were tachycardic and tachypneic. The PCV was higher in non-survivors (median = 39 (IQR = 12)%) compared to survivors (median = 37.5 (IQR = 7)%), but above the reference range (*p* = 0.009). A hyperlactatemia was noted in both groups with non-survivors (median = 2.4 (IQR = 3.5) mmol/L) higher than survivors (median = 1.5 (IQR = 1.9) mmol/L; *p* < 0.001). The alactic base excess was higher in the non-survivors (median = 5.0 (IQR = 9.3)) versus survivors (median = 3.9 (IQR = 8.10); *p* = 0.029), with a higher creatinine (median = 1.3 (IQR = 0.7) mg/dl vs. median = 1.2 (IQR = 0.45) mg/dl; *p* = 0.001), but an azotemia was not observed in either group. Hypocalcemia was present in both categories and was lower in the non-survivors (median = 5.3 (IQR = 0.5) mg/dl) versus survivors (median = 5.6 (IQR = 0.5) mg/dl; *p* < 0.001). The chloride was lower in the non-survivors compared to survivors as well (median = 101(IQR = 6.0) mg/dl vs. median = 102 (IQR = 4.0) mg/dl; *p* = 0.004) but was not out of reference range. Hyperglycemia was noted in both groups, with non-survivors (median = 151 (IQR = 91) mg/dl) higher than the survivors (122 (IQR = 65) mg/dl; *p* = 0.003).

**Table 3:** Univariate analysis for clinical variables associated with surgical survival.

Variable	Survival median (IQR) *N* = 125	Mortality median (IQR) *N* = 91	Wilcoxon test *p-*value
Age (years, *N* = 216)	12 (10)	15 (9)	0.01
Temperature (°F, *N* = 185)	99.6 (1.5)	99.4 (2.3)	0.293
Heart rate (beats/min, *N* = 205)	52 (22)	60 (32)	0.002
Respiratory rate (respirations/min, *N* = 197)	20 (12)	24 (12)	0.038
PCV (%, *N* = 189)	37.5 (7)	39 (12)	0.009
Total solids (g/dl, *N* = 188)	6.7 (0.9)	6.9 (1.2)	0.132
pH (*N* = 216)	7.42 (0.07)	7.42 (0.08)	0.287
pCO_2_ (mmHg, *N* = 215)	39.7 (9.85)	40.7 (13.1)	0.219
Base excess-ECF (mmol/L, *N* = 216)	1.5 (9.0)	1.0 (11.4)	0.767
Lactate (mmol/L, *N* = 216)	1.5 (1.9)	2.4 (3.5)	<0.001
Alactic base excess (mmol/L, *N* = 216)	3.9 (8.10)	5.0 (9.3)	0.029
HCO_3_ (mmol/L, *N* = 216)	26.4 (8.6)	25.7 (10.2)	0.670
Sodium (mmol/L, *N* = 216)	137 (3.0)	137 (3.0)	0.511
Potassium (mmol/L, *N* = 216)	3.6 (0.4)	3.5 (0.4)	0.771
Ionized calcium (mg/dL, *N* = 216)	5.6 (0.5)	5.3 (0.5)	<0.001
Ionized magnesium (mg/dL, *N* = 216)	1.0 (0.3)	1.0 (0.3)	0.086
Chloride (mmol/L, *N* = 216)	102 (4.0)	101 (6.0)	0.004
Anion gap (mmol/L, *N* = 212)	12.0 (7.0)	13.0 (11.0)	0.183
Strong ion difference (*N* = 216)	42.8 (4.3)	42.1 (5.3)	0.928
Creatinine (mg/dl, *N* = 213)	1.2 (0.45)	1.30 (0.7)	0.001
Blood urea nitrogen (mg/dl, *N* = 215)	16 (6.0)	17 (6.0)	0.174
Atot (*N* = 189)	1.34 (0.18)	1.38 (0.23)	0.165
Glucose (mg/dl, *N* = 216)	122 (65)	151 (91)	0.003

### 3.5 Univariate model performance

**Figure 1:**
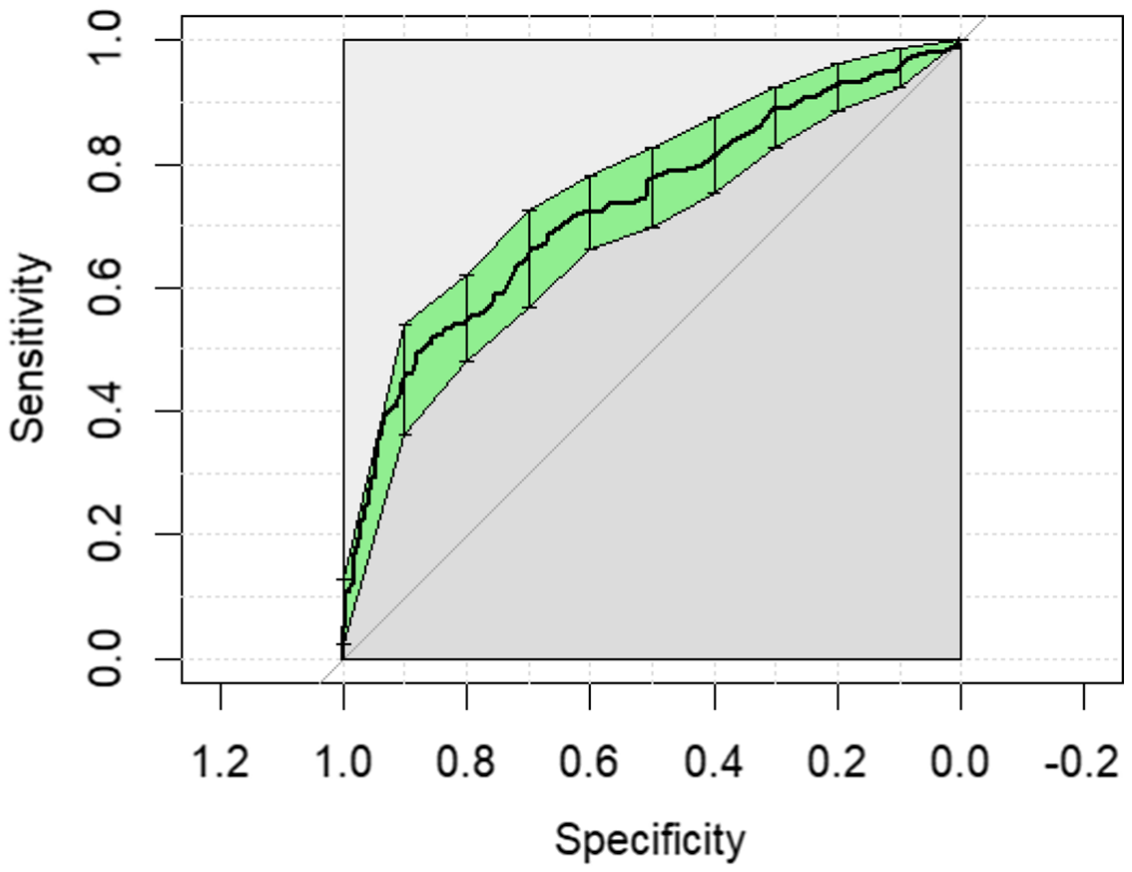
The univariate analysis receiver operator curve for the predictive value of serum glucose for medical or surgical treatment of horses with colic. The area under the curve was 0.729 (95% CI: 0.685–0.773).

**Figure 2:**
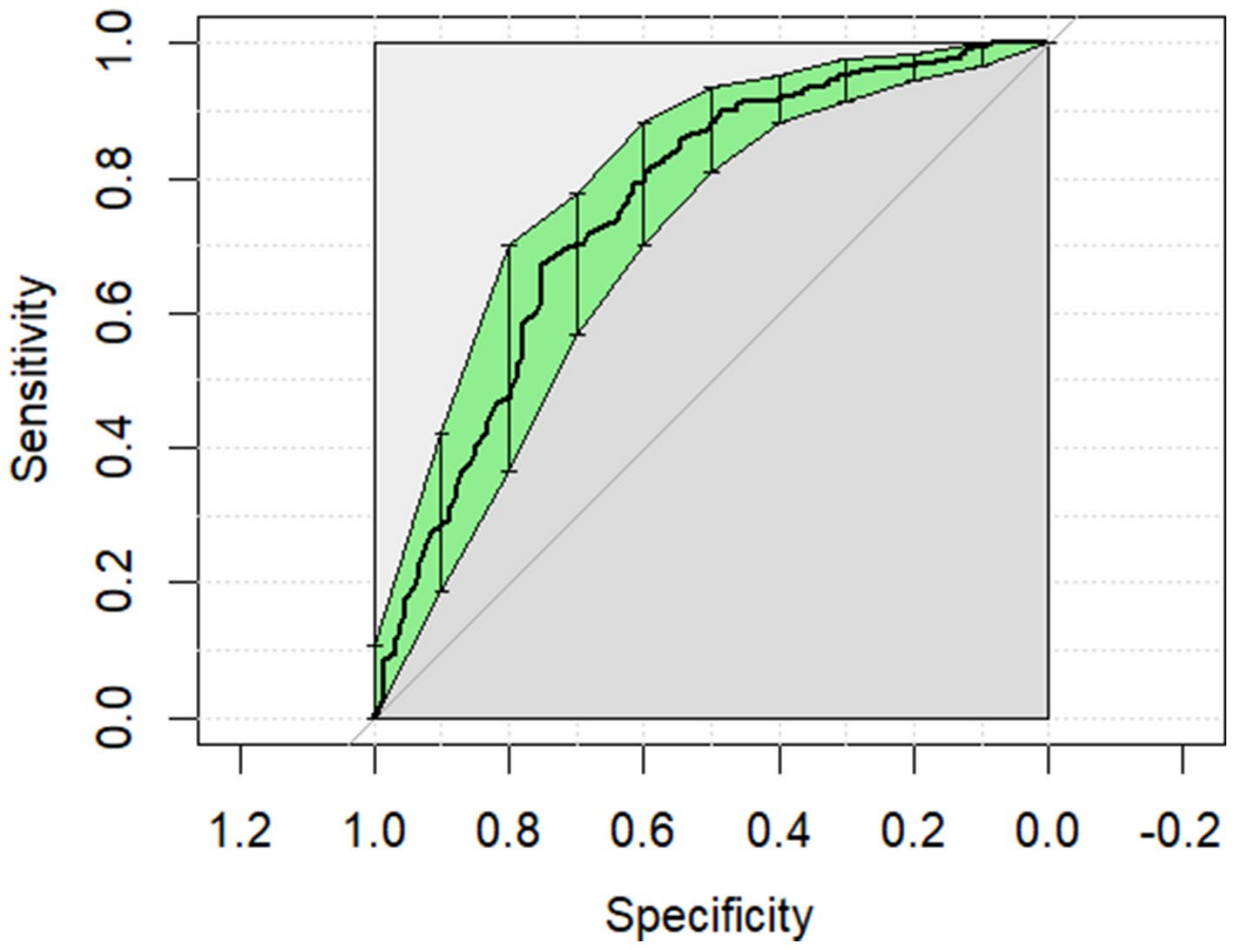
The univariate analysis receiver operator curve for the predictive value of serum glucose for strangulating or non-strangulating lesions in horses with colic. The area under the curve was 0.756 (95% CI: 0.706–0.806).

**Figure 3:**
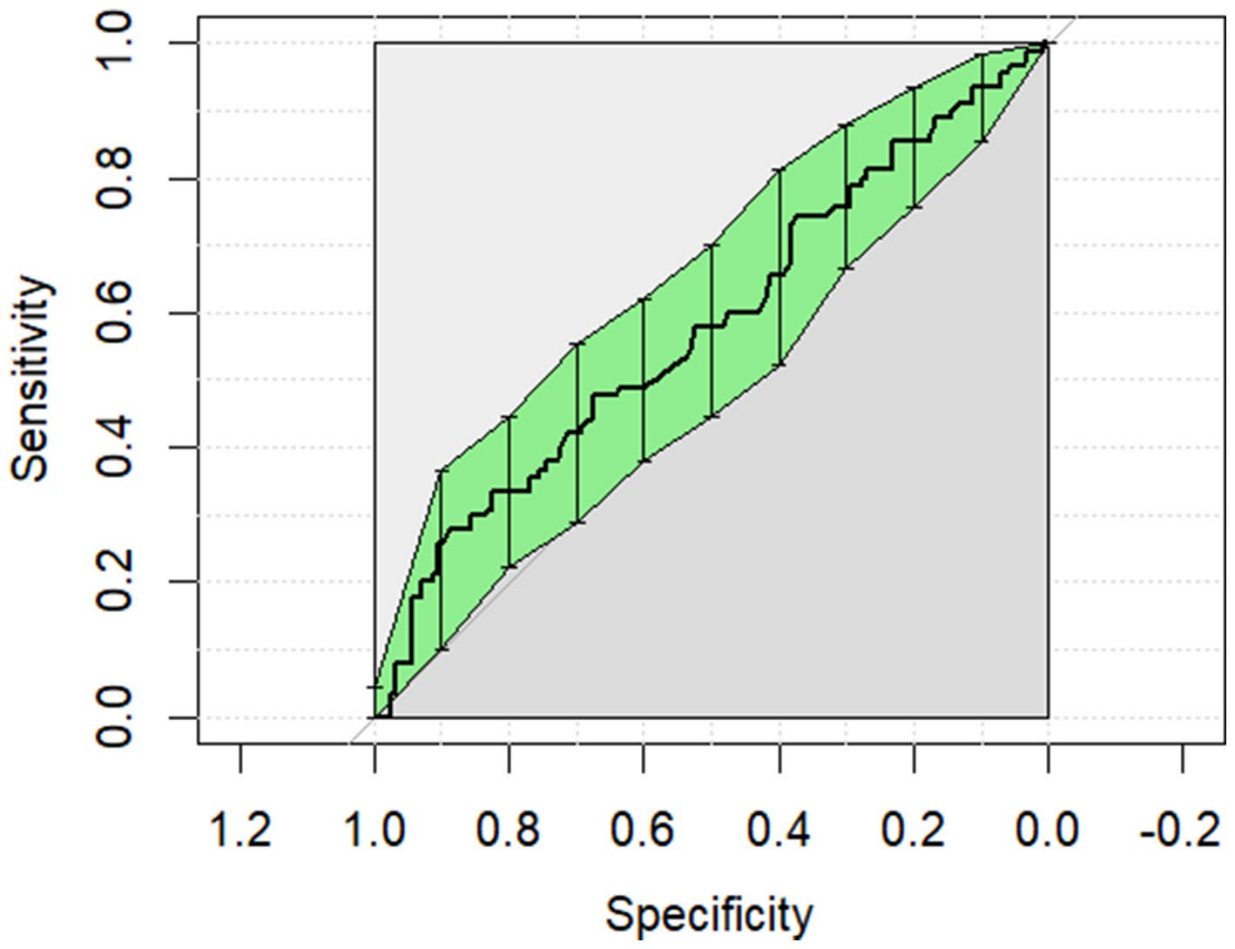
The univariate analysis receiver operator curve for the predictive value of alactic base excess for survival of surgical cases to discharge. The area under the curve was 0.587 (95% CI: 0.509–0.665).

The ROC curves generated for each category for the univariate analysis were of limited clinical utility. No single predictor had an AUC higher than 0.8. Glucose levels demonstrated the highest AUC, with comparisons of non-surgical to surgical cases reaching 0.729 (Figure 1) and comparisons of strangulating versus non-strangulating calculated at 0.756 (Figure 2). The AUC for alactic base excess ranged from 0.512–0.587 (Table 4 and Figure 3).

**Table 4:** Area under the curve for the receiver operator characteristic (AUC ROC) for individual variables to discriminate surgical versus non-surgical, strangulating obstruction (SO) versus non-SO and survival after surgery.

Variable	Surgical versus non-surgical	Strangulating versus non-strangulating	Survival versus mortality after surgery
Age	0.537	0.601	0.602
Temperature	0.602	0.692	0.546
Heart rate	0.667	0.653	0.627
Respiratory rate	0.591	0.596	0.586
PCV	0.519	0.457	0.611
Total solids	0.530	0.472	0.564
pH	0.602	0.604	0.458
pCO_2_	0.523	0.558	0.549
Base excess-ECF	0.546	0.512	0.488
Lactate	0.653	0.645	0.649
Alactic base excess	0.512	0.549	0.587
HCO_3_	0.535	0.501	0.483
Sodium	0.471	0.519	0.526
Potassium	0.509	0.524	0.512
Ionized calcium	0.663	0.680	0.649
Ionized magnesium	0.493	0.513	0.432
Chloride	0.583	0.605	0.615
Anion gap	0.576	0.551	0.554
Strong ion difference	0.556	0.526	0.504
Creatinine	0.575	0.461	0.631
Blood urea nitrogen	0.563	0.564	0.554
Atot	0.533	0.473	0.559
Glucose	0.729	0.756	0.618

The clinical meaningful threshold was set at 0.8. Only serum glucose passed the minimum threshold for surgical versus non-surgical cases, and for strangulating versus non-strangulating lesions.

### 3.6 Multi-variate analysis and modeling

The confusion matrix results of the Lasso analysis are presented based on the optimal cut-off (Table 5). The AUC was calculated for each comparison group. For medical versus surgical, it was 0.76 (95% CI = 0.71–0.81); for strangulating versus non-strangulating, it was 0.81 (95% CI = 0.76–0.86); and for survival to surgery, it was 0.76 (95% CI = 0.69–0.83). The AUC was less than the point estimate for all but strangulating versus non-strangulating (11). Alactic base excess was not a selected variable in any of the final models. Glucose was the largest coefficient in the models for medical versus surgical and for strangulating versus non-strangulating (Table 5). The other variables, though selected by lasso models, had little influence on the predicting probabilities based on the size of the coefficients. For survival from surgery, age and L-lactate levels had the largest coefficients.

**Table 5:** Multi-variate lasso analysis confusion matrix.

Variable	True positive	False positive	True negative	False negative
Surgical versus non-surgical (total *N* = 493; surgical *N* = 183, non-surgical *N* = 310)	139	101	209	44
Strangulating versus non-strangulating (total *N* = 493; strangulating *N* = 112 non-strangulating *N* = 381)	76	63	318	36
Survival versus mortality after surgery (total *N* = 183. survived *N* = 105, died *N* = 78)	57	32	73	21

Variable	Surgical versus non-surgical (*n* = 493)	Strangulating versus non-strangulating (*n* = 493)	Survival versus mortality after surgery (*n* = 183)
Selected variables (coefficient)	pH (−0.08) Ionized Calcium (−0.12) Glucose (0.63)	Glucose (0.55)	Age (0.21) Lactate (0.32) Ionized Calcium (−0.14) Ionized magnesium (−0.001) Chloride (−0.04) PCV (0.09)
Optimal cut-off for Y	0.30	0.25	0.38
Accuracy	0.71 (95% CI: 0.66–0.75)	0.80 (95% CI: 0.76–0.83)	0.71 (95% CI: 0.64–0.77)
Precision	0.58 (95% CI: 0.51–0.64)	0.54 (95% CI: 0.46–0.63)	0.64 (95% CI: 0.53–0.74)
Sensitivity	0.76 (95% CI: 0.69–0.82)	0.67 (95% CI: 0.58–0.76)	0.73 (95% CI: 0.62–0.82)
Specificity	0.67 (95% CI: 0.62–0.72)	0.83 (95% CI: 0.79–0.87)	0.69 (95% CI: 0.59–0.78)
AUC	0.76 (95% CI: 0.71–0.81)	0.81 (95% CI: 0.76–0.86)	0.76 (95% CI: 0.69–0.83)

AUC: area under the curve; PCV: packed cell volume; Y=Youden’s index.

## 4 Discussion

Alactic base excess has been introduced into human medical literature in an effort to distinguish metabolic acidosis secondary to an accumulation of lactic acid from a retention of fixed acids, including phosphoric acids and ketoacids. The correlation between ABE and mortality and as an indicator of renal insufficiency suggests ABE is a valuable diagnostic tool in critically ill patients. Prerenal azotemia is common in horses with colic, secondary to dehydration and fluid sequestration (3). Concurrent use of nephrotoxic medications including non-steroidal anti-inflammatory drugs increases the risk of renal damage, and development of acute kidney injury. However, current biomarkers including serum creatinine and azotemia are unable to predict the development of renal injury in horses (12, 13). In humans, negative ABE (less than –3 mmol/L) has been shown to predict renal injury, and the risk of mortality in septic patients, associated with the presence of acute kidney injury (5, 14). Conversely, a positive ABE was associated with an appropriate renal response, with a metabolic alkalosis secondary to volume contraction (5, 7). Regardless of the presence of renal failure, ABE was able to identify severe acidosis and predict mortality in humans (15).

The results of this study do not support the hypothesis of alactic base excess having value as a diagnostic tool, either individually or in combination with other metrics, that could be measured at admission and subsequently used to differentiate horses with medical or surgical colic, identify the presence of strangulating lesions or determine likelihood of survival after surgery. It was noted that horses that underwent exploratory celiotomy, particularly those that were non-survivors, did exhibit an elevated ABE (median 5 (9.3) mmol/L), compared to survivors (median 3.9 (8.1) mmol/L). Human patients with positive ABE values (>4 mmol/L) exhibit renal compensation to adjust for a metabolic acidosis with alternative compensatory mechanisms engaged in response to a decreasing pH (7). As pH remained in reference range and was not different between survivors and non-survivors in this study, it is possible that alterations in the acid–base balance were in flux due to the acute nature of the disease, or that the kidneys had fully compensated for any metabolic acidosis caused by the disease process.

For both surgical versus non-surgical comparisons, as well as for the diagnosis of a strangulating lesion, serum glucose was found to be the only parameter that remained in the final predictive models. Blood glucose concentrations have been described in the diagnosis of a strangulating lesions in the literature and were predictive in a model for identification of strangulating obstructions (16–18). Hyperglycemia has also been associated with a decreased chance of survival in horses that were presented for colic, but was not identified as a factor in our final model evaluating the survival of horses undergoing surgical intervention (16, 17, 19). Hyperglycemia is a stress response secondary to glycogenolysis and can be compounded by insulin dysregulation and preexisting equine metabolic disorders (20, 21). As survival was only assessed in this study a subset of the population with a surgical lesion, the severity of the disease process may have been a confounding factor.

In the final multivariate model, hyperlactatemia was associated with mortality in horses that underwent an exploratory celiotomy. Hyperlactatemia has been correlated with decreased likelihood for survival in those diagnosed with a large colon volvulus, with lower concentrations being associated with survival (22). In the cohort investigated by Johnston et al., horses that exhibited a plasma L-lactate concentration <6 mmol/L had a > 90% likelihood of survival to discharge, while the group with an L-lactate >7 mmol/L was found to have the chance of survival decreased to 30% (22). However, their study included both medical and surgical cases and did not focus on those that went to surgery. Delesalle et al. demonstrated with every 1 mmol increase in plasma and peritoneal L-lactate increased the likelihood of surgical intervention with a 1.3 times greater probability of non-survival (23). While L-lactate was not retained as a variable in our final multi-variate model for surgical versus non-surgical cases, the higher L-lactate in surgical cases, as well as hyperlactatemia in the non-survivors versus survivors is consistent with the previous literature. L-lactate is traditionally seen as an indicator of anaerobic metabolism, and other sources may include mitochondrial dysfunction, liver insufficiency, and the effects of catecholamines (24–28). Therefore, L-lactate contributes to acid base status but is not the sole determinant. Metabolic derangements can be further defined by ABE, which removes L-lactate from the calculation. As ABE was also elevated in these cases, it indicates that the source of increased L-lactate in these horses was likely due to hypovolemia (5).

In this study, increasing age was associated with a decreased chance of survival to discharge in horses undergoing exploratory celiotomy. The literature is conflicting regarding age and survival to discharge for horses experiencing colic. Southwood et al. delineated a correlation between a decreased overall short-term survival of geriatric (<16 years of age) compared to mature horses (between 4 and 15 years of age) (29). When this report is further examined, surgically treated colics showed no significant difference between geriatric and mature horses, except for those horses greater than 20 years of age (29). A more recent study demonstrated no significant influence of age on short-term survival post-operatively, however decreased long-term survival was understandably associated with increasing age in these horses (30). The association between age and survival may be affected by both owner perception and use of the horse, as the reason for euthanasia was not examined in our study.

A limitation of this study was that collection of venous blood gas samples is routinely obtained at this institution with the use of a manually heparinized syringe. This likely accounts for the hypocalcemia noted across all cohorts in this study, as heparin chelates calcium within the sample, falsely lowering the reported value (31). This error could have been circumvented with the use of non-heparinized syringes or citrate-titrated heparinized blood gas syringes, which do not have this effect (31, 32). As a referral institution, the majority of horses presented by owners to the hospital are admitted with the immediate submission of a venous blood gas. However, there may have been horses that did not receive bloodwork that were not included. In addition, some variables, including vital parameters and TS, were excluded due to lack of consistent recording, whereas others, such as PCO_2_, were excluded due to equipment failure leading to high numbers of missing values.

Additional limitations of this study include the retrospective nature, which may have introduced selection bias. As ABE has not yet been evaluated in veterinary literature, the number of cases may have been insufficient to assess the value of ABE in prognosis. A prospective multicenter investigation may be needed. While some colic patients become septic in their course of treatment, they do not often present as septic, which is a limitation in the application of ABE in horses with colic. Not all cases received follow up bloodwork to assess renal function or were euthanized early in case management, preventing evaluation of the usefulness of ABE in identification of renal dysfunction. However, as many horses present in varying states of dehydration and azotemia, renal function and injury caused by nephrotoxic medications in period prior to tertiary evaluation warrants investigation. Finally, the selection of medical or surgical treatment as well as euthanasia were based on the attending clinician’s recommendation and client consent. Emotional as well as financial reasons could have influenced the choice for surgical or medical management or euthanasia and affected the outcomes. An additional limitation is the exclusion of horses euthanized at the time of presentation, which were excluded from the study as they did not receive treatment.

In conclusion, while increased ABE was noted to be associated with mortality in horses undergoing surgical intervention, it was not selected in the final multivariate predictive models. Similar to other reports, increased serum glucose was associated with the presence of a strangulating surgical lesion and surgical intervention, and age was associated with mortality in horses undergoing surgery for colic. While the hypothesis was rejected, the usefulness of ABE in veterinary medicine requires additional investigation. As ABE is a reflection of acid base status and renal compensation, future investigations may focus on identification of patients with risk of acute kidney insufficiency or failure. These could include evaluation of septic neonates, due to their immature renal function. ABE could also prove useful in horses with enterocolitis, as their acid base derangements are often complex. Treatment of diarrhea, specifically Potomac horse fever, requires the use of nephrotoxic antimicrobials, further increasing the risk of subclinical to fulminant renal injury. As ABE has been shown to be an early predictor of renal dysfunction in humans, it may prove to be a useful tool for identification of renal injury in equine medicine.

## Data Availability

The raw data supporting the conclusions of this article will be made available by the authors, without undue reservation.
