# Supplementary material for: Alactic base excess is not a sensitive or specific diagnostic tool for outcome in horses with colic
Source: Front Vet Sci. 2025 Aug 15;12:1618304. doi: 10.3389/fvets.2025.1618304 (PMC12396145; doi:10.3389/fvets.2025.1618304)
Supplement: Supplementary file 1 [file Table_1.docx]

**Supplementary Table 1:** Reference intervals for vital parameters and blood gas variables.

| Variable | Reference Interval |
| --- | --- |
| Temperature (°F)* | 99.5-101.5 |
| Heart Rate (beats/minute)* | 25-45 |
| Respiratory Rate (respirations/minute)* | 10-15 |
| PCV(%)* | 35-46 |
| Total Solids (g/dl)* | 6.3-7.0 |
| pH | 7.34-7.44 |
| pCO_2_ (mmHg) | 38.9-53.9 |
| Base Excess-ECF (mmol/L) | 1.5-10.6 |
| Lactate (mmol/L) | 0.3-1.1 |
| Alactic Base Excess (mmol/L): calculation | -3.0-4.0 |
| HCO_3_ (mmol/L) | 26.9-35.5 |
| Sodium (mmol/L) | 132-140 |
| Potassium (mmol/L) | 2.6-5.0 |
| Ionized Calcium (mg/dL) | 6.1-7.1 |
| Ionized Magnesium (mg/dL) | 1.0-1.3 |
| Chloride (mmol/L) | 99-107 |
| Anion Gap (mmol/L): calculation | 1.5-11.5 |
| Strong Ion Difference (mmol/L): calculation | 38-42 |
| Creatinine (mg/dl) | 0.6-2.1 |
| Blood Urea Nitrogen (mg/dl) | 12-25 |
| Atot (mmol/L): calculation | 12-13mmol/L |
| Glucose (mg/dl) | 78-105 |

*Saunders Equine Formulary & Clinical Biochemistry of Domestic Animals
